# Are price discounts on sugar-sweetened beverages (SSB) linked to household SSB purchases? – a cross-sectional study in a large US household and retail scanner database

**DOI:** 10.1186/s12937-021-00673-w

**Published:** 2021-03-14

**Authors:** Yichen Zhong, Amy H. Auchincloss, Mark F. Stehr, Brent A. Langellier

**Affiliations:** 1 Department of Epidemiology and Biostatistics, Dornsife School of Public Health, Drexel University, Philadelphia, PA 19104 USA; 2 School of Economics, LeBow College of Business, Drexel University, Philadelphia, PA 19104 USA; 3 Department of Health Management and Policy, Dornsife School of Public Health, Drexel University, Philadelphia, PA 19104 USA

**Keywords:** Marketing, Nutrition, Socioeconomic factors, Sugar-sweetened beverages, Price promotion

## Abstract

**Background:**

Price promotions on sugar-sweetened beverages (SSBs) are commonly used by retailers to provide economic incentives for purchasing. Surprisingly, there is a lack of high-quality articles that examine the frequency and magnitude of sugary beverage discounting and consumer responses to discounts. The objective of this study is to quantify the association between exposure to price discounts and SSB purchases.

**Methods:**

This cross-sectional study linked 2016 SSB consumption data from a U.S. household consumer panel (analytic sample *N* = 11,299 households) and weekly prices at stores where they shopped. We derived percent of the time SSBs were discounted (annual promotion frequency) and the amount of the discount (annual promotion magnitude) and assessed their association with household annual per capita SSB purchase ounces. Linear regression models adjusted for household size, income per capita, age, education, presence of children, race, occupation, region, and urbanicity. We also evaluated whether the association between promotion and purchase varied by socioeconomic status and race subgroups. Data were analyzed in 2019–2020.

**Results:**

On average, households were exposed to SSBs price promotions 44% of the time. A 10-percentage point increase in annual SSB promotion frequency was associated with 13.7% increase in annual per capita purchasing (*P* < 0.0001), and a 1-percentage point increase in annual SSB promotion magnitude was associated with 15.3% increase in annual per capita purchasing (*P* < 0.0001). These associations did no vary significantly across socioeconomic status and race subgroups (Interaction *P* > 0.2).

**Conclusions:**

More frequent and deeper price promotion was associated with higher annual per capita SSB purchases. Restricting SSB price promotions may be effective at reducing SSB consumption.

**Supplementary Information:**

The online version contains supplementary material available at 10.1186/s12937-021-00673-w.

## Background

Decreasing the consumption of sugar-sweetened beverages (SSB) in the population can reduce the burden of obesity, type 2 diabetes, hypertension, cardiovascular diseases and other health conditions [1, 2]. Previous research suggests that SSB purchases may be responsive to price changes [3]. Price promotion, or temporary price reduction, is commonly used to provide an economic incentive for purchasing. For shelf stable and storable products like SSBs, price promotions not only increase near-term consumption, but also lead to stockpiling, which may lead to increased consumption in the long-term [4].

Literature on the prevalence of food and beverage price promotion in US stores is limited. A case study in an urban supermarket in the US documented that SSBs were on sale for one-third of the year [5]. Another study collected in-store data throughout the US and found prevalence of promotion of SSBs in supermarkets was higher relative to other beverages [6]. Other studies examined promotion of food and beverages in Australia, Netherlands, and United Kingdom (UK) and also found high prevalence of price promotion [7–10]. A systematic review found that consumers take advantage of price discounts to purchase a high proportion (ranging from 29 to 56%) of these products on promotion [11].

Regulating or restricting price promotion of SSBs has been proposed as a policy option to reduce the consumption of SSBs in England, Scotland, and California in the US [12–14]. However, surprisingly little research has been done to quantify the association between SSB price promotion and SSB purchases or consumption in the population. One study in the UK of a demographically representative sample of 30,000 British households found that price promotions increased total purchase of SSBs by 31% [15].

Numerous studies have found that higher consumption of SSBs among low-income and minority populations parallels these groups’ higher burdens of diet related chronic disease [16–19]. Differences in exposure or responsiveness to SSB price promotion frequency and magnitude may contribute to inequities in diet and chronic disease. Though further research is needed, a handful of studies have found that own-price elasticities of various foods are higher among lower-income population than others, suggesting that it is plausible that lower-income populations would be particularly responsive to price promotions [20]. However, little is known about the impact of SSB price promotion on purchases and consumption by socioeconomic status and race/ethnicity.

We used a large US household panel and retail scanner database to examine the association between the annual promotion frequency and magnitude experienced at the household level and household annual SSB purchases. We hypothesized that increased exposure to price promotion would be associated with more SSB purchases and that responsiveness to price promotion would vary by socioeconomic status and race.

## Methods

This is a cross-sectional study using data from the 2016 Nielsen Consumer Panel Database (Household Panel) and the Nielsen Retail Scanner Database (Retailer Data).

### Household panel data

The Household Panel data contains longitudinal household purchasing data from a large nationally representative household sample [21]. The study design and data collection were described in detail elsewhere [22]. Briefly, the panelist was expected to record the date and the store for each shopping trip, and then scan all purchases for in-house use. Data collection included: purchasing date, retailer, product identification (i.e., universal product code – UPC), quantity, and prices. Panelists’ demographic, socioeconomic status and geographic location were also collected.

### Retailer data

The Retailer Data contains weekly transaction data from approximately 35,000 individual stores from about 90 retail chains in designated marketing areas throughout the US [23]. In general, the marketing areas coincided with the Household Panel’s location. The Retailer Data were primarily collected from grocery stores, chain convenience stores, drug stores, and mass merchandisers. The weekly transaction data in the Retailer Data included UPC codes and price. SSBs were identified using a Product Module Code available in the Retailer Data, details in Table S1. Retailer discounts and specials, including loyalty card or retailer coupons, were factored into the store’s weekly price; manufacturer coupons were not captured in the price.

### Study sample

The Household Panel data for each shopping trip were linked with the Retailer Data by common retailer identification codes for each store. The Retailer Data contained weekly transaction data that we needed to determine SSB price promotion frequency and magnitude, the main exposures in this study. We retained only households who purchased most (≥ 80%) of their SSB purchases from stores in the Retailer Data. This restriction ensured that the stores were representative of the retail environment where the households shopped for SSBs. The analytic sample was 11,299 households which was 19% of the total Household Panel (11,299/60,169, selection flow diagram is shown in Fig. S1). Sensitivity analyses compared results when sample inclusion criteria were defined as purchased ≥80% of SSBs from stores in Retailer Data vs. purchased ≥ 60%, ≥ 70%, or ≥ 90% of SSBs from stores in the Retailer Data.

### Exposure definition

Two variables, *annual promotion magnitude* and *annual promotion frequency*, were used to approximate the level of SSB price promotion in stores where each household shopped during the year (details in Supplement S2). These exposure variables were determined by weekly store prices in relation to the modal prices (i.e., most frequently observed prices) of SSB products. We defined exposure to price promotion based on promotions at all store(s) that each household shopped at during the year.

### Outcome definition

The outcome was annual per capita SSB purchase ounces (*annual per* capita *purchase*), calculated as a household’s total SSB purchase ounces in 2016 divided by the household size. The outcome variables were aggregated for the whole year of 2016 to eliminate the impact of seasonal trends. Similar approaches have been used in previous research on price promotion and purchases [15, 24, 25]. SSBs included in this study can be categorized as carbonated soft drinks and fruit drinks. Carbonated soft drinks were primarily regular soda. Fruit drinks were fruit-flavored, non-carbonated drink with 0–50% fruit juice. Note that the purchase volume excludes non-caloric diet beverages and 100% fruit juice.

### Covariates

It is well-documented that consumers’ demographic and socioeconomic characteristics are related to beverage consumption and responses to price promotion [15, 26], thus, analyses adjusted for all relevant covariates available in the Household Panel data. The Household Panel collected information on household size and income (from which we derived income per capita [mid-point of household income categories / household size]; presence of children; age, education, and occupation class of male and female heads of household; and race (white, black, other, based on the ‘racial identity’ of the household), Hispanic origin (based on whether members of the household are of Hispanic origin), region, and urbanicity (based on county rural-urban continuum codes) [27].

### Statistical analysis

Due to the skewness of the annual per capita purchase, descriptive tables present medians (and 25th–75th percentiles) and the variable was log-transformed in regression analyses. The exposure variables were roughly normally distributed.

Ordinary least squares regressions were used to evaluate the association between annual promotion frequency/magnitude and the log-transformed annual per capita purchase adjusted for household size, household income per capita, male head age, female head age, male head education, female head education, presence of children, race, male head occupation, female head occupation, region, and urban/rural setting (regression model specification is presented in the supplement, Table S3). Annual promotion frequency was multiplied by 10 in the regression analysis, so the exponentiated coefficient can be interpreted as the percent change in annual per capita purchase related to a 10 percentage points increase in annual promotion frequency. Annual promotion magnitude was multiplied by 100 in the regression analysis, so the exponentiated coefficient can be interpreted as the percent change in annual per capita purchase related to a 1 percentage point increase in annual promotion magnitude. In addition, interaction terms were used to evaluate whether the association between annual price promotion and annual per capita purchase varied by socioeconomic status and race subgroups. All data were analyzed in 2019–2020.

## Results

The household characteristics are summarized by annual per capita purchase quartile in Table 1. Approximately 50% of the household heads were 50+ years. Households with higher SSB purchase tended to have lower income, lower male and female head education, male head blue collar occupation, and identified as black race.

**Table 1:** Baseline household characteristics by annual per capita purchase of sugar-sweetened beverages

	Annual Per Capita Purchase				Total
	Quartile 1	Quartile 2	Quartile 3	Quartile 4	
	< 153 oz	153 to < 496 oz	496 to < 1394 oz	≥1394 oz	
**Sample size**	2824	2825	2825	2825	11,299
**Household size, N (%)**					
1 Member	796 (28.2)	718 (25.4)	631 (22.3)	859 (30.4)	3004 (26.6)
2 Members	1212 (42.9)	1154 (40.8)	1195 (42.3)	1231 (43.6)	4792 (42.4)
3/4 Members	631 (22.3)	736 (26.1)	787 (27.9)	616 (21.8)	2770 (24.5)
5+ Members	185 (6.6)	217 (7.7)	212 (7.5)	119 (4.2)	733 (6.5)
**Household Income, N (%)**					
< $25,000	293 (10.4)	276 (9.8)	324 (11.5)	495 (17.5)	1388 (12.3)
$25,000 - $34,000	248 (8.8)	247 (8.7)	287 (10.2)	380 (13.5)	1162 (10.3)
$35,000 - $49,000	462 (16.4)	431 (15.3)	437 (15.5)	509 (18.0)	1839 (16.3)
$50,000 - $69,000	514 (18.2)	546 (19.3)	522 (18.5)	532 (18.8)	2114 (18.7)
$70,000 - $99,000	603 (21.4)	680 (24.1)	649 (23.0)	551 (19.5)	2483 (22.0)
≥ $100,000	704 (24.9)	645 (22.8)	606 (21.5)	358 (12.7)	2313 (20.5)
**Income per capita, N (%)**					
≤ $15,000	398 (14.1)	471 (16.7)	558 (19.8)	652 (23.1)	2079 (18.4)
$15,001 - $30,000	1036 (36.7)	1034 (36.6)	1037 (36.7)	1046 (37.0)	4153 (36.8)
$30,001 - $50,000	768 (27.2)	731 (25.9)	707 (25.0)	676 (23.9)	2882 (25.5)
≥ $50,000	622 (22.0)	589 (20.8)	523 (18.5)	451 (16.0)	2185 (19.3)
**Male head age, N (%)**					
< 35 Years	175 (6.2)	226 (8.0)	199 (7.0)	130 (4.6)	730 (6.5)
35–54 Years	674 (23.9)	765 (27.1)	795 (28.1)	730 (25.8)	2964 (26.2)
55+ Years	1188 (42.1)	1097 (38.8)	1135 (40.2)	1225 (43.4)	4645 (41.1)
No Male Head	787 (27.9)	737 (26.1)	696 (24.6)	740 (26.2)	2960 (26.2)
**Female head age, N (%)**					
< 35 Years	275 (9.7)	321 (11.4)	288 (10.2)	190 (6.7)	1074 (9.5)
35–54 Years	849 (30.1)	939 (33.2)	1035 (36.6)	875 (31.0)	3698 (32.7)
55+ Years	1420 (50.3)	1301 (46.1)	1249 (44.2)	1326 (46.9)	5296 (46.9)
No Female Head	280 (9.9)	264 (9.3)	253 (9.0)	434 (15.4)	1231 (10.9)
**Male head education, N (%)**					
High School or Less	419 (14.8)	455 (16.1)	576 (20.4)	742 (26.3)	2192 (19.4)
Some College	479 (17.0)	548 (19.4)	592 (21.0)	642 (22.7)	2261 (20.0)
College Grad	1139 (40.3)	1085 (38.4)	961 (34.0)	701 (24.8)	3886 (34.4)
No Male Head	787 (27.9)	737 (26.1)	696 (24.6)	740 (26.2)	2960 (26.2)
**Female head education, N (%)**					
High School or Less	482 (17.1)	458 (16.2)	575 (20.4)	749 (26.5)	2264 (20.0)
Some College	669 (23.7)	732 (25.9)	766 (27.1)	762 (27.0)	2929 (25.9)
College Grad	1393 (49.3)	1371 (48.5)	1231 (43.6)	880 (31.2)	4875 (43.1)
No Female Head	280 (9.9)	264 (9.3)	253 (9.0)	434 (15.4)	1231 (10.9)
**Male head occupation, N (%)**					
White Collar	1041 (36.9)	988 (35.0)	882 (31.2)	715 (25.3)	3626 (32.1)
Blue Collar	391 (13.8)	471 (16.7)	612 (21.7)	661 (23.4)	2135 (18.9)
Other	605 (21.4)	629 (22.3)	635 (22.5)	709 (25.1)	2578 (22.8)
No Male Head	787 (27.9)	737 (26.1)	696 (24.6)	740 (26.2)	2960 (26.2)
**Female head occupation, N (%)**					
White Collar	1282 (45.4)	1329 (47.0)	1324 (46.9)	1085 (38.4)	5020 (44.4)
Blue Collar	200 (7.1)	241 (8.5)	273 (9.7)	267 (9.5)	981 (8.7)
Other	1062 (37.6)	991 (35.1)	975 (34.5)	1039 (36.8)	4067 (36.0)
No Female Head	280 (9.9)	264 (9.3)	253 (9.0)	434 (15.4)	1231 (10.9)
**Presence of children, N (%)**					
No	2271 (80.4)	2161 (76.5)	2122 (75.1)	2354 (83.3)	8908 (78.8)
Yes	553 (19.6)	664 (23.5)	703 (24.9)	471 (16.7)	2391 (21.2)
**Race, N (%)**					
White	2414 (85.5)	2373 (84.0)	2329 (82.4)	2347 (83.1)	9463 (83.8)
Black	142 (5.0)	199 (7.0)	279 (9.9)	279 (9.9)	899 (8.0)
Other	268 (9.5)	253 (9.0)	217 (7.7)	199 (7.0)	937 (8.3)
**Hispanic origin, N (%)**					
No	2685 (95.1)	2643 (93.6)	2621 (92.8)	2678 (94.8)	10,627 (94.1)
Yes	139 (4.9)	182 (6.4)	204 (7.2)	147 (5.2)	672 (5.9)
**Region, N (%)**					
New England	179 (6.3)	171 (6.1)	201 (7.1)	152 (5.4)	703 (6.2)
Middle Atlantic	183 (6.5)	167 (5.9)	134 (4.7)	105 (3.7)	589 (5.2)
East North Central	461 (16.3)	465 (16.5)	479 (17.0)	525 (18.6)	1930 (17.1)
West North Central	213 (7.5)	209 (7.4)	194 (6.9)	218 (7.7)	834 (7.4)
South Atlantic	455 (16.1)	535 (18.9)	527 (18.7)	562 (19.9)	2079 (18.4)
East South Central	148 (5.2)	164 (5.8)	212 (7.5)	223 (7.9)	747 (6.6)
West South Central	220 (7.8)	228 (8.1)	247 (8.7)	232 (8.2)	927 (8.2)
Mountain	352 (12.5)	370 (13.1)	379 (13.4)	420 (14.9)	1521 (13.5)
Pacific	613 (21.7)	516 (18.3)	452 (16.0)	388 (13.7)	1969 (17.4)
**Metropolitan/urban/rural** ^**a**^**, N (%)**					
Metropolitan	2588 (91.6)	2575 (91.2)	2545 (90.1)	2487 (88.0)	10,195 (90.2)
Urban and Rural	236 (8.4)	250 (8.8)	280 (9.9)	338 (12.0)	1104 (9.8)

^a^Metropolitan: population more than 250,000; Urban and rural: population less than 250,000

The 11,299 households in the analytic sample made 223,096 SSB purchases from 7500 stores in the 2016 Retailer Data. All purchases included were made from food, drug and mass merchandiser retail channels. Further details on store types were not available in the Retailer Data. Most of these included stores were food stores and in metropolitan areas (Table S4).

The annual household per-capita SSB purchase by beverage category and price promotion quartiles are summarized in Table 2. Annual per capita median purchase was 496 oz (based on 12-oz servings this translates to 0.8 serving per week). Households who shopped in stores with higher promotion frequency or magnitude purchased more SSBs. For example, households who shopped in stores with low promotion frequency (1st quartile) purchased a median of 331 oz per capita per year (0.53 servings per week) vs. households who shopped for stores with high promotion frequency (4th quartile) purchased a median of 670 oz per capita per year (1.07 servings per week). Carbonated soft drinks were the most popular beverages, followed by fruit drinks. The most popular package size was 12 oz, 12 pack (416 oz), followed by 2-liter bottle (195 oz).

**Table 2:** Summary of annual per capita purchase of sugar-sweetened beverages

	Annual per capita purchase	
	Mean ounces (STD)	Median ounces (IQR)
**Beverage category** ^**a**^		
Overall	1251.7 (2242.6)	495.6 (153.0, 1394.9)
Carbonated soft drinks	932.6 (2047.9)	244.0 (48.0, 904.9)
Fruit drinks	319.2 (630.4)	103.1 (23.0, 348.4)
**Exposure to price promotion described as annual promotion frequency** ^**b**^		
Quartile 1, < 27% time experienced promotion	991.8 (1922.3)	330.7 (94.0, 1069.9)
Quartile 2, 27 to < 46%	1178.2 (2166.4)	460.9 (135.2, 1301.0)
Quartile 3, 46 to < 62%	1265.8 (2128.4)	545.0 (190.0, 1454.2)
Quartile 4, ≥ 62%	1566.3 (2645.2)	670.1 (250.9, 1739.5)
**Exposure to price promotion described as annual promotion magnitude** ^**c**^		
Quartile 1, experienced < 3.8% off the modal price	968.9 (1899.6)	306.7 (88.3, 1009.9)
Quartile 2, 3.0 to < 4.9% off	1169.7 (2225.8)	444.5 (140.1, 1273.4)
Quartile 3, 4.9 to < 5.8% off	1301.0 (2111.1)	580.8 (195.2, 1522.3)
Quartile 4, ≥ 5.8% off	1567.3 (2629.3)	673.2 (246.4, 1752.0)

^a^Beverage categories were defined using the Produce Module Description (“Carbonated soft drinks” and “Fruit drinks”) in the Retailer Database. Diet beverages and 100% juice were excluded using information from UPC Description, Product Description, Formula Description, and Type Description in the Retailer Database. “Carbonated soft drinks” were primarily regular soda. “Fruit drinks” (AKA non-carbonated juice drinks, juice beverages, fruit cocktails, or fruit flavored drinks) were fruit-flavored, non-carbonated drinks with 0–50% fruit juice. Note that the dataset did not contain an indicator for energy drinks, thus, they were included in carbonated (e.g., Red Bull, Monster) and fruit drink (e.g., Gatorade) categories.

^b^Annual promotion frequency was calculated as: $\frac{Weeks\ experiencing\ge 5\% magnitude\ of\ price\ promotion\ for\ the\ household}{Number\ of\ weeks\ in\ 2016.}$

^c^Annual promotion magnitude was calculated as: $\frac{\sum_{i=1}^{number\ of\ weeks\ in\ 2016} Promotion\ magnitude\ for\ the\ household\ in\ week\ i}{Number\ of\ weeks\ in\ 2016}$

Table 3 describes the annual promotion frequency and magnitude the households experienced in 2016. Overall, at least one of the stores at which households shopped during the year had price promotions for SSBs 44% of the time, and the average annual price was approximately 95% (1 – 0.046 = 0.954) of the modal price. SSB promotion frequency and magnitude were higher in stores frequented by larger households, households with children, those who were middle-income (based on per capita income), higher educated (at least some college), and non-White. There was large variation in SSB promotion frequency and magnitude by geographic region and urban/rural setting. Households in the New England region experienced the lowest price promotion, and households in Pacific and West South Central regions had the largest price promotion. Households in metropolitan or densely populated urban settings experienced more frequent and deeper price promotion than households in less populated urban or rural settings.

**Table 3:** Summary of annual price promotion experienced by households in the sample, by household characteristics and region

	Annual Promotion Frequency ^**a**^, Mean (STD)	Annual Promotion Magnitude ^**b**^, Mean (STD)
**Overall**	0.444 (0.238)	0.046 (0.021)
**Household size**		
1 Member	0.429 (0.234)	0.045 (0.021)
2 Members	0.437 (0.243)	0.045 (0.022)
3/4 Members	0.466 (0.235)	0.048 (0.020)
5+ Members	0.463 (0.233)	0.048 (0.021)
**Household Income**		
< $25,000	0.421 (0.231)	0.045 (0.021)
$25,000 - $34,000	0.441 (0.239)	0.046 (0.020)
$35,000 - $49,000	0.438 (0.231)	0.046 (0.020)
$50,000 - $69,000	0.445 (0.237)	0.046 (0.021)
$70,000 - $99,000	0.452 (0.242)	0.046 (0.022)
≥ $100,000	0.453 (0.245)	0.047 (0.021)
**Income per capita**		
≤ $15,000	0.438 (0.234)	0.046 (0.020)
$15,001 - $30,000	0.449 (0.237)	0.046 (0.021)
$30,001 - $50,000	0.446 (0.240)	0.046 (0.022)
≥ $50,000	0.436 (0.243)	0.045 (0.021)
**Male head age**		
< 35 Years	0.463 (0.228)	0.048 (0.019)
35–54 Years	0.460 (0.237)	0.047 (0.021)
55+ Years	0.434 (0.241)	0.045 (0.022)
No Male Head	0.438 (0.237)	0.046 (0.021)
**Female head age**		
< 35 Years	0.466 (0.223)	0.048 (0.019)
35–54 Years	0.454 (0.241)	0.047 (0.022)
55+ Years	0.434 (0.240)	0.045 (0.021)
No Female Head	0.434 (0.235)	0.046 (0.020)
**Male head education**		
High School or Less	0.435 (0.244)	0.045 (0.023)
Some College	0.452 (0.229)	0.047 (0.020)
College Grad	0.448 (0.241)	0.046 (0.021)
No Male Head	0.438 (0.237)	0.046 (0.021)
**Female head education**		
High School or Less	0.433 (0.242)	0.045 (0.022)
Some College	0.449 (0.236)	0.047 (0.021)
College Grad	0.448 (0.239)	0.046 (0.021)
No Female Head	0.434 (0.235)	0.046 (0.020)
**Male head occupation**		
White Collar	0.458 (0.240)	0.047 (0.021)
Blue Collar	0.443 (0.240)	0.046 (0.022)
Other	0.431 (0.235)	0.045 (0.021)
No Male Head	0.438 (0.237)	0.046 (0.021)
**Female head occupation**		
White Collar	0.448 (0.237)	0.046 (0.021)
Blue Collar	0.447 (0.242)	0.046 (0.023)
Other	0.441 (0.240)	0.046 (0.021)
No Female Head	0.434 (0.235)	0.046 (0.020)
**Presence of children**		
No	0.437 (0.239)	0.045 (0.021)
Yes	0.470 (0.234)	0.048 (0.020)
**Race**		
White	0.437 (0.238)	0.045 (0.022)
Black	0.482 (0.245)	0.050 (0.019)
Other	0.474 (0.232)	0.049 (0.018)
**Hispanic origin**		
No	0.442 (0.239)	0.046 (0.021)
Yes	0.476 (0.222)	0.049 (0.018)
**Region**		
New England	0.241 (0.240)	0.030 (0.024)
Middle Atlantic	0.428 (0.247)	0.044 (0.020)
East North Central	0.466 (0.200)	0.048 (0.019)
West North Central	0.467 (0.275)	0.041 (0.036)
South Atlantic	0.387 (0.279)	0.043 (0.020)
East South Central	0.394 (0.199)	0.047 (0.018)
West South Central	0.508 (0.203)	0.051 (0.015)
Mountain	0.494 (0.190)	0.049 (0.018)
Pacific	0.498 (0.213)	0.051 (0.015)
**Metropolitan/urban/rural**^**3**^		
Metropolitan	0.452 (0.238)	0.047 (0.020)
Urban and Rural	0.364 (0.230)	0.038 (0.025)

^a^Annual promotion frequency was calculated as: $\frac{Weeks\ experiencing\ge 5\% magnitude\ of\ price\ promotion\ for\ the\ household}{Number\ of\ weeks\ in\ 2016}$.

^b^Annual promotion magnitude was calculated as: $\frac{\sum_{i=1}^{number\ of\ weeks\ in\ 2016} Promotion\ magnitude\ for\ the\ household\ in\ week\ i}{Number\ of\ weeks\ in\ 2016}$.

^c^Metropolitan: population more than 250,000; Urban and rural: population less than 250,000.

The association between SSB price promotion and annual per capita purchase after adjusting for baseline household characteristics is shown in Table 4. Overall, for every 10 percentage point increase in the annual promotion frequency, there was a 13.7% increase in annual per capita purchases (*P* < 0.0001, Section A), and for every 1 percentage point increase in annual promotion magnitude there was a 15.3% increase in per capita purchases (*P <* 0.0001). For example, an increase from < 27% to ≥ 62% annual promotion frequency (roughly equivalent to the difference between the top quartile and bottom quartile) was associated with a 48% increase in annual per capita purchases; an increase from < 3.8% to ≥ 5.8% annual promotion magnitude (roughly equivalent to the difference between the top quartile and bottom quartile) was associated with a 30.6% increase in annual per capita purchases. These findings are consistent with the hypothesis that increased exposure to price promotion would be associated with more SSB purchases. The association between SSB price promotion and annual per capita purchase was similar across socioeconomic status and race subgroups (Type-3 *P* for interactions > 0.2, Table 4 Section B).

**Table 4:** Association between sugar sweetened beverages annual price promotion and per capita purchase ^a^

	Association between annual promotion frequency × 10 and annual per capita purchase ^**d**^		Association between annual promotion magnitude × 100 and annual per capita purchase ^**e**^	
**Section A. Overall association between price promotion and per capita purchase**				
	Exp Estimate (95% CI)	*P*-value	Exp Estimate (95% CI)	*P-*value
**Overall**	1.137 (1.123–1.151)	*<0.0001*	1.153 (1.137–1.169)	*<0.0001*
**Section B. Association between price promotion and per capita purchase stratified by income per capita, female head education and race**				
	Exp Estimate (95% CI)	Interaction *P-*value	Exp Estimate (95% CI)	Interaction *P-*value
**Income per capita** ^**b**^				
≤ $15,000	1.127 (1.096–1.160)	*0.7777*	1.138 (1.102–1.175)	*0.7517*
$15,001 - $30,000	1.144 (1.122–1.167)		1.156 (1.131–1.182)	
$30,001 - $50,000	1.141 (1.115–1.168)		1.162 (1.132–1.193)	
≥ $50,000	1.129 (1.100–1.159)		1.147 (1.112–1.182)	
**Female head education** ^**b,c**^				
High School or Less	1.148 (1.119–1.179)	*0.4783*	1.168 (1.135–1.201)	*0.2954*
Some College	1.139 (1.113–1.166)		1.147 (1.117–1.178)	
College Grad	1.127 (1.106–1.147)		1.141 (1.118–1.165)	
No Female Head	1.157 (1.116–1.199)		1.186 (1.137–1.237)	
**Race** ^**b**^				
White	1.138 (1.122–1.153)	*0.9963*	1.148 (1.131–1.165)	*0.2625*
Black	1.138 (1.093–1.185)		1.191 (1.129–1.257)	
Other	1.135 (1.089–1.184)		1.184 (1.121–1.250)	

^a^Ordinary least squares linear regression was used. The outcome, annual per capita purchase, was log transformed. Models adjusted for household size, household income per capita, male head age, female head age, male head education, female head education, presence of children, race, male head occupation, female head occupation, region, and urban/rural setting.

^b^Estimates were obtained using the main effect (e.g., promotion magnitude) and interaction terms.

^c^Education was specific to female-head and male-head. Female head education was selected as a socioeconomic status indicator because women are typically the primary grocery shoppers and food preparers. (Citation: Crane et al. Nutr Educ Behav. 2019;51 (2):199–204.)

^d^Annual promotion frequency was calculated as: $\frac{Weeks\ experiencing\ge 5\% magnitude\ of\ price\ promotion\ for\ the\ household}{Number\ of\ weeks\ in\ 2016}$. An exponentiated coefficient of 1.137 can be interpreted as 10 percentage points increase in annual promotion frequency is associated with 13.7% higher annual per capita purchase.

^e^Annual promotion magnitude was calculated as: $\frac{\sum_{i=1}^{number\ of\ weeks\ in\ 2016} Promotion\ magnitude\ for\ the\ household\ in\ week\ i}{Number\ of\ weeks\ in\ 2016}$. An exponentiated coefficient of 1.153 can be interpreted as a 1 percentage point increase in annual promotion magnitude is associated with 15.3% higher annual per capita purchase.

Figure S2 and S3 show that the results were robust to alternate definitions of household weekly price promotion exposure (yes/no) based on the magnitude of the promotion in that week (main results show price promotion defined as weekly price promotion magnitude ≥ 5% vs. sensitivity analysis: ≥ 10%, ≥ 15%), and alternate sample inclusion criteria (main analysis: purchased ≥ 80% SSBs from stores in the Retailer Data vs. sensitivity analysis: ≥ 60%, ≥ 70%, and ≥ 90%). Results from sensitivity analyses using an alternative definition of weekly price promotion magnitude experienced by the household (an intermediate step in calculating the household annual price promotion exposure, see Supplement S2 for details) is presented in Table S5. In the main analysis, the weekly price promotion magnitude for a household was determined by the largest discount observed in that week among stores where they purchased SSBs in 2016. This definition was based on the premise that seeing a large discount would create a stronger incentive for purchasing compared to a small or no discount [28]. In the sensitivity analysis, instead of the largest discount, average discount in that week among stores they shopped in 2016 was used. With the alternative definition, larger annual promotion magnitude was still associated with significantly higher annual per capita purchase, but the association was attenuated compared with the main analysis.

## Discussion

This cross-sectional study using a large nationally representative household sample found that SSBs were price-promoted 44% of the time, and the average annual price was discounted approximately 5%. SSB price promotion was positively associated with annual per capita SSB purchases. Every 10 percentage points increase in the percent of weeks SSBs were discounted during the year was associated with 13.7% increase in SSB purchases (*P* < 0.0001). Every 1 percentage point increase in the annual promotion magnitude was associated with 15.3% increase in SSB purchases (*P <* 0.0001).

Consumption of SSBs in the US started to decline in recent years [26, 29]. However, the prevalence of obesity is still high and SSBs remains a major source of calories and added sugars in the population diet [26, 30]. Price levers, especially SSB taxes, are increasingly used to reduce SSB consumptions worldwide [31]. Restricting price promotions presents an alternative policy lever of potential importance. Price promotions incentivize purchases for short-term consumption and create a perceived need to seize the temporary cost-saving opportunity which can lead to stockpiling (e.g., purchase for future). The latter incentive cannot be addressed by other economic strategies such as soda taxes or floor prices. California recently attempted to prohibit beverage companies from providing incentives or financial support to distributors or retailers for promotions [14]. Governments in UK and Scotland are trying to restrict food and beverages with high fat, sugar and salt [12, 13]. However, the lack of high-quality evidence base supporting the effectiveness of restricting price promotions in reducing SSB consumption has been noted as a major barrier for adopting such policy [32].

The present study adds to the extremely limited literature reporting the SSB price promotion prevalence and magnitude in the US and the potential impact on consumption. Our results show that price promotion is being used aggressively by retailers (likely in conjunction with manufacturers [33]) to incentivize sugary beverage purchases and consumers respond strongly to these incentives. A number of studies have described prevalence of retail SSB promotions, though few have directly assessed how price promotions affect household SSB purchasing. Prevalence of price promotion for foods and beverages has ranged from 24 to 67% across studies [11]. Our finding that, on average, at least one of the stores where households shopped were price promoting SSBs 44% of the time, falls in the middle of this range. The only other large study to examine the effect of SSB price promotion on households’ SSB purchasing was a large demographically representative household sample from the UK, which found that price promotion was associated with a 31% increase in SSB purchase [15]. Our results also suggested large impact of price promotion on SSB purchase: an increase from < 27% to ≥ 62% annual promotion frequency (roughly equivalent to the difference between the top quartile and bottom quartile) was associated with a 48% increase in annual per capita purchases.

Our study suggested that reducing the exposure to price promotion may be effective in reducing SSB consumption in the population. Further empirical interventional studies are needed to evaluate the causal impact of an intervention restricting price promotion. In addition, strategies need to be developed to address the anticipated public resistance and industry opposition to a policy targeting price promotion. Dietary habits are difficult to change, and it is unrealistic to expect a single intervention to achieve the desired impact. It is likely that a suite of interventions will be needed. Economic interventions (e.g., restricting price promotion, beverage tax, floor price) and behavioral interventions (e.g., increasing nutritional knowledge, promoting proper dietary behaviors) can be considered.

### Strengths and limitations

This study has several strengths, particularly that little is known about the effect of SSB price promotions on households’ SSB purchasing in the US. First, the study was conducted in a large and geographically representative sample of households in the US. Second, we determined price promotion using weekly pricing data from the Retailer Data, which have wide geographic coverage and include mass merchandise, supermarkets, grocery stores, and drug stores (stores were classified by retail channel, but detailed store types were not available) [23]. The price promotion exposure was determined objectively by store weekly prices and was less prone to recall bias. Third, we standardized the magnitude of SSB promotion across stores using weights based on volume purchased in the household sample. This standardization made the price promotion variables comparable across stores and households. However, we were less able to describe the purchasing pattern in response to promotion of products with small market share (e.g., private label beverages). Fourth, the exposure (price promotion) was measured independently from the outcome (actual household purchases). Defining exposure in this way helped mitigate potential confounding that could occur when defining exposure to price promotion based on the actual stores where households shopped on a given trip; specifically, households may select stores with deep price promotions on SSB for trips in which they intend to purchase large quantities of SSBs. The association between SSB price promotion and purchase observed in this study assumed that households’ purchases co-occurred with retailer price promotions. This assumption is reasonable based on immediate and drastic increase in demand during promotion found in other studies [34, 35].

This study also has several limitations. First, with a cross-sectional design, we were not able to determine whether a change in price promotion exposure would lead to a change in purchases. Second, the sample had low SSB consumption. Households in our sample purchased 0.8 serving per capita per week (or 16 cal per capita per day based on calories from one serving of a 12 oz. can of regular coke). This is similar to volume of SSB purchases in major SSB categories (regular carbonated soft drinks and fruit/sports/energy drinks) in another study that used Nielsen’s household panel [36]. However, a national survey reported averages that were at least 75 SSB calories per day [26]. Though we lack data to directly ascertain the reason for lower consumption in the Nielsen data, the purchasing data did not include SSBs purchased in restaurants, vending machines, or other food away from home outlets. Nevertheless, SES variations in SSB consumption found in other work were maintained in this sample (for example, households with higher SSB purchase were of lower socioeconomic status) thus offering rough face validity for within sample comparisons [26]. Third, we retained only households who purchased most of their SSBs from stores in the Retailer Data so we can determine price promotion exposure using store prices. It is unlikely that this exclusion had a large impact on results. Sensitivity analyses found results were robust to alternate purchase thresholds. Furthermore – although excluded households had lower socioeconomic status and purchased slightly more SSBs (Appendix Table S6 shows characteristics for households who purchased any SSB in the study period) – our results found no evidence that the association between price promotion and SSB purchase varied by socioeconomic status.

## Conclusions

More frequent and deeper price promotion on SSBs was associated with higher household annual per capita purchase of SSBs. The strong association between price promotion and annual per capita purchase observed in our study adds to the limited evidence base and suggests that restricting price promotion on SSBs might be effective in reducing purchase, consumption, and potentially related disease burden.

## Supplementary Information

**Additional file 1: Table S1.** Product Module Codes for included sugar-sweetened beverages. **Supplementary Notes S2.** Derivations of annual promotion magnitude and frequency at the household level. **Table S3.** Specification of the regression models. **Table S4.** Store characteristics, retailer database. **Table S5.** Sensitivity analysis - association between household annual price promotion and per capita purchase. Results using alternate definition of weekly price promotion magnitude experienced by the household – average promotion magnitude among stores they shopped in 2016, instead of the largest promotion among the stores in the main analysis. **Table S6.** Baseline household characteristics for households who purchased any sugar-sweetened beverages during the study period, by annual per capita purchase. **Fig. S1.** Sample Selection. **Fig. S2.** Sensitivity analysis. Comparison of results when weekly SSB price promotion for each household is defined as experiencing a weekly price magnitude X ≥ 5% vs. ≥ 2%, ≥ 10%, ≥ 15%. **Fig. S3.** Sensitivity analysis. Comparison of results when sample inclusion criteria is defined as purchased ≥ 80% of SSBs from stores in retailer database vs. purchased Y ≥ 60%, ≥ 70%, or ≥ 90% of SSBs from stores in the retailer database.

## Data Availability

The data that support the findings of this study are available from the Nielsen Company (US), LLC but restrictions apply to the availability of these data, which were used under license for the current study, and so are not publicly available.

## Abbreviations

SSB: Sugar-sweetened beverages
UK: United Kingdom
Household Panel: Nielsen Consumer Panel Database
Retailer Data: Nielsen Retail Scanner Database
UPC: Universal Product Code
