# Supplementary material for: Are price discounts on sugar-sweetened beverages (SSB) linked to household SSB purchases? – a cross-sectional study in a large US household and retail scanner database
Source: Nutr J. 2021 Mar 14;20:29. doi: 10.1186/s12937-021-00673-w (PMC7980678; doi:10.1186/s12937-021-00673-w)
Supplement: Supplementary file 1 — Additional file 1: Table S1. Product Module Codes for included sugar-sweetened beverages. Supplementary Notes S2. Derivations of annual promotion magnitude and frequency at the household level. Table S3. Specification of the regression models. Table S4. Store characteristics, retailer database. Table S5. Sensitivity analysis - association between household annual price promotion and per capita purchase. Results using alternate definition of weekly price promotion magnitude experienced by the household – average promotion magnitude among stores they shopped in 2016, instead of the largest promotion among the stores in the main analysis. Table S6. Baseline household characteristics for households who purchased any sugar-sweetened beverages during the study period, by annual per capita purchase. Fig. S1. Sample Selection. Fig. S2. Sensitivity analysis. Comparison of results when weekly SSB price promotion for each household is defined as experiencing a weekly price magnitude X ≥ 5% vs. ≥ 2%, ≥ 10%, ≥ 15%. Fig. S3. Sensitivity analysis. Comparison of results when sample inclusion criteria is defined as purchased ≥ 80% of SSBs from stores in retailer database vs. purchased Y ≥ 60%, ≥ 70%, or ≥ 90% of SSBs from stores in the retailer database. [file 12937_2021_673_MOESM1_ESM.docx]

## Supplementary File

[Table S1. Product Module Codes for included sugar-sweetened beverages 2](#_Toc63078744)

[Supplementary Notes S2. Derivations of annual promotion magnitude and frequency at the household level 3](#_Toc63078745)

[Table S3. Specification of the regression models 5](#_Toc63078746)

[Table S4. Store characteristics, retailer database 6](#_Toc63078747)

[Table S5. Sensitivity analysis - association between household annual price promotion and per capita purchase. Results using alternate definition of weekly price promotion magnitude experienced by the household – average promotion magnitude among stores they shopped in 2016, instead of the largest promotion among the stores in the main analysis 7](#_Toc63078748)

[Table S6. Baseline household characteristics for households who purchased any sugar-sweetened beverages during the study period, by annual per capita purchase 8](#_Toc63078749)

[Figure S1. Sample Selection 10](#_Toc63078750)

[Figure S2. Sensitivity analysis. Comparison of results when weekly SSB price promotion for each household is defined as experiencing a weekly price magnitude X ≥5% vs. ≥2%, ≥10%, ≥15% 11](#_Toc63078751)

[Figure S3. Sensitivity analysis. Comparison of results when sample inclusion criteria is defined as purchased ≥80% of SSBs from stores in retailer database vs. purchased Y≥60%, ≥70%, or ≥90% of SSBs from stores in the retailer database 12](#_Toc63078752)

Table S1. Product Module Codes for included sugar-sweetened beverages ^a^

| **Product Module Code** | **Product Module Description** |
| --- | --- |
| 1030 | Fruit drinks & juices |
| 1042 | Fruit drinks - other container |
| 1484 | Soft drinks - carbonated |

Notes:

^a^ Diet beverages and 100% juice were excluded using information from UPC Description, Product Description, Formula Description, and Type Description in the Scanner Database.

Supplementary Notes S2. Derivations of annual promotion magnitude and frequency at the household level

Two variables, *annual promotion magnitude* and *annual promotion frequency*, were used to approximate the level of SSB price promotion in stores where each household shopped during the year. These exposure variables were measured using weekly store prices and were independent of the actual purchases by the households. Thus, these variables reflect the promotion frequency and magnitude in stores. Details follow below.

*Annual promotion magnitude*

First, promotion magnitude was calculated for each UPC in each store each week. It was calculated as the ratio of the price in a given store in a given week (numerator) to its modal price (denominator, i.e., most frequently observed price) in this store during the year.

Second, promotion magnitude was calculated for the SSB category overall for each store each week. In order to make this measure comparable across stores (and not overly influenced by UPCs that were rarely purchased), we weighted the category-level promotion magnitude based on each UPC’s volume purchased in the 2016 Household Panel. In other words, the SSBs price promotion magnitude was purchase-volume weighted to reflect the discounts on products with more volume purchased in the sample. Purchases of 10,310 UPCs were used to determine the weights. The weighted price promotion magnitude for the SSB category for each store each week was calculated as $1-\sum_{i=1}^{N} r_{i}w_{i}$, where$N$ is the total number of UPCs (i.e., 10,310); $i$ denotes individual UPC; $r$ is the ratio of the weekly price for the UPC vs. the modal price for this UPC in the store (calculated in the first step); and $w$ is the weight based on annual volume purchased in the sample. For a UPC with zero sales in a store in a given week, we assumed no price promotion for this UPC.

Third, the promotion magnitude for a household in each week was determined by the largest discount in that week observed among the stores where they purchased SSBs in 2016. Defining promotion magnitude using the largest discount was based on the premise that seeing a large discount would create a stronger incentive for purchasing compared to a small or no discount.^1^ Consumers may obtain information about retailer price promotions in a variety of ways that cannot be distinguished in this study, for example on-shelf displays/call-outs ($1 off, loyalty card 10% off, buy 2 get 1 free, etc.) or retailer circulars.^2^ In sensitivity analyses, we also tested an alternate definition, the average of price promotion magnitude among the stores in a certain week.

Finally, the exposure, *annual promotion magnitude*, was calculated for each household as the mean of weekly price promotion magnitude throughout the year.

*Annual promotion frequency*

We derived another exposure variable, *annual promotion frequency*, using a similar approach. We first created a binary price promotion variable (yes/no) for each household each week and defined it as promotion magnitude for the SSB category ≥0.05 (or ≥5%) for this household in this week. The 5% threshold has been used by others in prior work^3,4^ and in our dataset is the 50th percentile of the distribution of *annual promotion magnitude*. Sensitivity analyses evaluated the binary price promotion variables defined using smaller (i.e., ≥2%) and larger (i.e., ≥10% and ≥15%) weekly SSB price promotion magnitudes. *Annual promotion frequency* for each household was then estimated as percent of weeks with price promotion during the year.

Table S3. Specification of the regression models

Equation 1. Ordinary least squares regressions evaluating the association between annual price promotion magnitude and annual per capita purchase

$$\log(Annual per capita purchase)=\alpha_{0}+\beta_{1}(Annual Promotion Magnitude\times100)+\sum_{i=2}^{N} \beta_{i}\mathrm{Cov}_{i}$$

$\mathrm{Cov}_{i}$ are confounders including panelists’ demographic, social-economic and geographic characteristics, household composition, etc.

$exp(\beta_{1})$ can be interpreted as the % increase in annual per capita purchase associated every 1 percentage point in annual promotion magnitude

Equation 2. Ordinary least squares regressions evaluating the association between annual price promotion frequency and annual per capita purchase

$$\log(Annual per capita purchase)=\alpha_{0}+\beta_{1}(Annual Promotion Frequency\times10)+\sum_{i=2}^{N} \beta_{i}\mathrm{Cov}_{i}$$

$exp(\beta_{1})$ can be interpreted as the % increase in annual per capita purchase associated every 10 percentage points in annual promotion frequency

Equation 3. Ordinary least squares regressions evaluating whether the association between annual price promotion frequency and annual per capita purchase varies in socioeconomic and race subgroups

$$\log(Annual per capita purchase)=\alpha_{0}+\beta_{1}Subgroup+\beta_{2}\left( Annual Promotion Frequency\times10 \right)+\beta_{3}Subgroup\times\left( Annual Promotion Frequency\times10 \right) +\sum_{i=4}^{N} \beta_{i}{Cov}_{i}$$

Subgroups tested: income per capita, female head education, and race.

Equation 4. Ordinary least squares regressions evaluating whether the association between annual price promotion magnitude and annual per capita purchase varies in socioeconomic and race subgroups

$\log(Annual per capita purchase)=\alpha_{0}+\beta_{1}Subgroup+\beta_{2}\left( Annual Promotion \mathrm{Magnitude}\times100 \right)+\beta_{3}Subgroup\times\left( Annual Promotion \mathrm{Magnitude}\times100 \right) +\sum_{i=4}^{N} \beta_{i}{Cov}_{i}$

Table S4. Store characteristics, retailer database

| **Store characteristics** | | N=7,500 |
| --- | --- | --- |
| **Channel, N (%)** | |  |
|  | Drug | 1,017 (13.6) |
|  | Food | 5,380 (71.7) |
|  | Mass merchandiser | 1,103 (14.7) |
| **Urban/Rural, N (%)** | |  |
|  | Metro - population more than 1,000,000 | 4,552 (60.7) |
|  | Metro - population 250,000 to 1,000,000 | 1,507 (20.1) |
|  | Metro - population less than 250,000 | 701 (9.3) |
|  | Urban - population more than 20,000, adjacent to a metro area | 297 (4.0) |
|  | Urban - population more than 20,000, not adjacent to a metro area | 120 (1.6) |
|  | Urban - population 2,500 to 9,999, adjacent to a metro area | 194 (2.6) |
|  | Urban - population 2,500 to 9,999, not adjacent to a metro area | 105 (1.4) |
|  | Rural - population less than 2,500, adjacent to a metro area | 11 (0.1) |
|  | Rural - population less than 2,500, not adjacent to a metro area | 12 (0.2) |
|  | Not available | 1 (0.0) |

Table S5. Sensitivity analysis - association between household annual price promotion and per capita purchase. ^a^ Results using alternate definition of weekly price promotion magnitude experienced by the household – average promotion magnitude among stores they shopped in 2016, instead of the largest promotion among the stores in the main analysis

| **Association between annual promotion frequency**$\boldsymbol{\times}$**10 and annual per capita purchase ^b^** | | | | **Association between annual promotion magnitude**$\boldsymbol{\times}$**100 and annual per capita purchase ^c^** | | | |
| --- | --- | --- | --- | --- | --- | --- | --- |
| Exp Coefficient (95% CI) | | P-value | | Exp Coefficient (95% CI) | | | P-value |
| 1.008 (0.995 - 1.022) | | 0.2333 | | 1.030 (1.015 - 1.045) | | | <.0001 |
|  |  | |  | |  |  |  |

Table Notes:

Around 90% of households purchased SSBs at multiple stores in 2016. As explained in the Methods, the promotion magnitude for a household in each week was determined by the largest discount in that week observed among the stores where they purchased SSBs in 2016. Defining promotion magnitude using the largest discount was based on the premise that seeing a large discount would create a stronger incentive for purchasing compared to a small or no discount. We estimated that more than 70% of SSBs were purchased from the store with the largest discount in the week among the stores each household shopped at during the year, which suggested that defining the price promotion exposure based on the largest discounts in the main analysis was a reasonable assumption (results not shown).

Table S5 shows results using an alternate definition, average promotion magnitude among stores they shopped in 2016 (instead of the largest promotion among the stores). With the alternative definition, larger annual promotion magnitude was still associated with significantly higher annual per capita purchase, but the association was attenuated compared with the main analysis. The association between annual price promotion frequency and annual per capita purchase was attenuated and p-values were>=0.2.

Table Footnotes:

^a^ Ordinary least squares linear regression was used. The outcome, annual per capita purchase, was log transformed. The models adjusted for household size, household income per capita, male head age, female head age, male head education, female head education, presence of children, race, male head occupation, female head occupation, region, and urban/rural setting.

^b^ Annual promotion frequency was calculated as: $\frac{Weeks experiencing \geq5\% magnitude of price promotion for the household}{Number of weeks in 2016}$. An exponentiated coefficient of 1.008 can be interpreted as 10 percentage points increase in annual promotion frequency is associated with 0.8% higher annual per capita purchase.

^c^ Annual promotion magnitude was calculated as: $\frac{\sum_{i=1}^{number of weeks in 2016} Promotion magnitude for the household in week i}{Number of weeks in 2016}$. An exponentiated coefficient of 1.030 can be interpreted as a 1 percentage point increase in annual promotion magnitude is associated with 3% higher annual per capita purchase.

**Table S6. Baseline household characteristics for households who purchased any sugar-sweetened beverages during the study period, by annual per capita purchase**

|  |  | **Annual Per Capita Purchase** | | | | | | **Total** |
| --- | --- | --- | --- | --- | --- | --- | --- | --- |
|  |  | **Quartile 1** | **Quartile 2** | | **Quartile 3** | | **Quartile 4** |  |
|  | | <245 oz | 245 to <681 oz | | 681 to <1716 oz | | ≥1716 oz |  |
| **Sample size** | | 14,679 | 14,681 | | 14,679 | | 14,690 | 58,719 |
| **Household size, N (%)** | |  |  | |  | |  |  |
|  | 1 Member | 3,329 (22.7) | 2,969 (20.2) | | 2,887 (19.7) | | 4,051 (27.6) | 13,236 (22.5) |
|  | 2 Members | 6,004 (40.9) | 5,818 (39.6) | | 6,015 (41.0) | | 6,401 (43.6) | 24,238 (41.3) |
|  | 3/4 Members | 3,971 (27.1) | 4,422 (30.1) | | 4,402 (30.0) | | 3,506 (23.9) | 16,301 (27.8) |
|  | 5+ Members | 1,375 (9.4) | 1,472 (10.0) | | 1,375 (9.4) | | 722 (4.9) | 4,944 (8.4) |
| **Household Income, N (%)** | | |  | |  | |  |  |
|  | < $25k | 1,648 (11.2) | 1,715 (11.7) | | 1,993 (13.6) | | 2,919 (19.9) | 8,275 (14.1) |
|  | $25 - $34k | 1,389 (9.5) | 1,535 (10.5) | | 1,672 (11.4) | | 2,011 (13.7) | 6,607 (11.3) |
|  | $35 - $49k | 2,380 (16.2) | 2,420 (16.5) | | 2,538 (17.3) | | 2,835 (19.3) | 10,173 (17.3) |
|  | $50 - $69k | 2,676 (18.2) | 2,700 (18.4) | | 2,712 (18.5) | | 2,721 (18.5) | 10,809 (18.4) |
|  | $70 - $99k | 3,286 (22.4) | 3,330 (22.7) | | 3,188 (21.7) | | 2,504 (17.1) | 12,308 (21.0) |
|  | $100k+ | 3,300 (22.5) | 2,981 (20.3) | | 2,576 (17.5) | | 1,690 (11.5) | 10,547 (18.0) |
| **Income per capita, N (%)** | | |  | |  | |  |  |
|  | <= $15,000 | 2,921 (19.9) | 3,282 (22.4) | | 3,681 (25.1) | | 4,017 (27.4) | 13,901 (23.7) |
|  | $15,001 - $30,000 | 5,632 (38.4) | 5,838 (39.8) | | 5,766 (39.3) | | 5,537 (37.7) | 22,773 (38.8) |
|  | $30,001 - $50,000 | 3,542 (24.1) | 3,295 (22.4) | | 3,248 (22.1) | | 3,261 (22.2) | 13,346 (22.7) |
|  | ≥$50,000 | 2,584 (17.6) | 2,266 (15.4) | | 1,984 (13.5) | | 1,865 (12.7) | 8,699 (14.8) |
| **Male head age, N (%)** | |  |  | |  | |  |  |
|  | < 35 Years | 1,048 (7.1) | 1,080 (7.4) | | 983 (6.7) | | 673 (4.6) | 3,784 (6.4) |
|  | 35-54 Years | 4,006 (27.3) | 4,338 (29.5) | | 4,438 (30.2) | | 3,974 (27.1) | 16,756 (28.5) |
|  | 55+ Years | 5,901 (40.2) | 5,681 (38.7) | | 5,775 (39.3) | | 6,053 (41.2) | 23,410 (39.9) |
|  | No Male Head | 3,724 (25.4) | 3,582 (24.4) | | 3,483 (23.7) | | 3,980 (27.1) | 14,769 (25.2) |
| **Female head age, N (%)** | |  |  | |  | |  |  |
|  | < 35 Years | 1,501 (10.2) | 1,560 (10.6) | | 1,406 (9.6) | | 996 (6.8) | 5,463 (9.3) |
|  | 35-54 Years | 4,981 (33.9) | 5,408 (36.8) | | 5,526 (37.6) | | 4,936 (33.6) | 20,851 (35.5) |
|  | 55+ Years | 7,024 (47.9) | 6,573 (44.8) | | 6,554 (44.6) | | 6,853 (46.7) | 27,004 (46.0) |
|  | No Female Head | 1,173 (8.0) | 1,140 (7.8) | | 1,193 (8.1) | | 1,895 (12.9) | 5,401 (9.2) |
| **Male head education, N (%)** | | | |  | |  | |  |
|  | High School or Less | 2,652 (18.1) | 3,151 (21.5) | | 3,684 (25.1) | | 4,381 (29.8) | 13,868 (23.6) |
|  | Some College | 2,920 (19.9) | 3,094 (21.1) | | 3,180 (21.7) | | 3,057 (20.8) | 12,251 (20.9) |
|  | College Grad | 5,383 (36.7) | 4,854 (33.1) | | 4,332 (29.5) | | 3,262 (22.2) | 17,831 (30.4) |
|  | No Male Head | 3,724 (25.4) | 3,582 (24.4) | | 3,483 (23.7) | | 3,980 (27.1) | 14,769 (25.2) |
| **Female head education, N (%)** | | | |  | |  | |  |
|  | High School or Less | 2,854 (19.4) | 3,174 (21.6) | | 3,710 (25.3) | | 4,505 (30.7) | 14,243 (24.3) |
|  | Some College | 3,716 (25.3) | 3,995 (27.2) | | 4,183 (28.5) | | 4,058 (27.6) | 15,952 (27.2) |
|  | College Grad | 6,936 (47.3) | 6,372 (43.4) | | 5,593 (38.1) | | 4,222 (28.8) | 23,123 (39.4) |
|  | No Female Head | 1,173 (8.0) | 1,140 (7.8) | | 1,193 (8.1) | | 1,895 (12.9) | 5,401 (9.2) |
| **Male head occupation, N (%)** | | | | | |  | |  |
|  | White Collar | 4,967 (33.8) | 4,597 (31.3) | | 4,239 (28.9) | | 3,231 (22.0) | 17,034 (29.0) |
|  | Blue Collar | 2,772 (18.9) | 3,317 (22.6) | | 3,653 (24.9) | | 3,865 (26.3) | 13,607 (23.2) |
|  | Other | 3,216 (21.9) | 3,185 (21.7) | | 3,304 (22.5) | | 3,604 (24.6) | 13,309 (22.7) |
|  | No Male Head | 3,724 (25.4) | 3,582 (24.4) | | 3,483 (23.7) | | 3,980 (27.1) | 14,769 (25.2) |
| **Female head occupation, N (%)** | | | |  | |  | |  |
|  | White Collar | 6,638 (45.2) | 6,627 (45.1) | | 6,257 (42.6) | | 5,364 (36.5) | 24,886 (42.4) |
|  | Blue Collar | 1,253 (8.5) | 1,421 (9.7) | | 1,548 (10.5) | | 1,615 (11.0) | 5,837 (9.9) |
|  | Other | 5,615 (38.3) | 5,493 (37.4) | | 5,681 (38.7) | | 5,806 (39.6) | 22,595 (38.5) |
|  | No Female Head | 1,173 (8.0) | 1,140 (7.8) | | 1,193 (8.1) | | 1,895 (12.9) | 5,401 (9.2) |
| **Presence of children, N (%)** | | | |  | |  | |  |
|  | No | 10,974 (74.8) | 10,482 (71.4) | | 10,537 (71.8) | | 12,013 (81.8) | 44,006 (74.9) |
|  | Yes | 3,705 (25.2) | 4,199 (28.6) | | 4,142 (28.2) | | 2,667 (18.2) | 14,713 (25.1) |
| **Race, N (%)** | |  |  | |  | |  |  |
|  | White | 12,284 (83.7) | 11,858 (80.8) | | 11,607 (79.1) | | 11,711 (79.8) | 47,460 (80.8) |
|  | Black | 1,006 (6.9) | 1,479 (10.1) | | 1,940 (13.2) | | 2,052 (14.0) | 6,477 (11.0) |
|  | Other | 1,389 (9.5) | 1,344 (9.2) | | 1,132 (7.7) | | 917 (6.2) | 4,782 (8.1) |
| **Hispanic origin, N (%)** | |  |  | |  | |  |  |
|  | No | 13,715 (93.4) | 13,583 (92.5) | | 13,585 (92.5) | | 13,845 (94.3) | 54,728 (93.2) |
|  | Yes | 964 (6.6) | 1,098 (7.5) | | 1,094 (7.5) | | 835 (5.7) | 3,991 (6.8) |
| **Region, N (%)** | |  |  | |  | |  |  |
|  | New England | 825 (5.6) | 737 (5.0) | | 649 (4.4) | | 545 (3.7) | 2,756 (4.7) |
|  | Middle Atlantic | 1,946 (13.3) | 1,946 (13.3) | | 1,876 (12.8) | | 1,667 (11.4) | 7,435 (12.7) |
|  | East North Central | 2,418 (16.5) | 2,556 (17.4) | | 2,595 (17.7) | | 2,815 (19.2) | 10,384 (17.7) |
|  | West North Central | 1,206 (8.2) | 1,142 (7.8) | | 1,163 (7.9) | | 1,279 (8.7) | 4,790 (8.2) |
|  | South Atlantic | 2,828 (19.3) | 3,002 (20.4) | | 3,099 (21.1) | | 3,213 (21.9) | 12,142 (20.7) |
|  | East South Central | 709 (4.8) | 895 (6.1) | | 1,000 (6.8) | | 1,192 (8.1) | 3,796 (6.5) |
|  | West South Central | 1,425 (9.7) | 1,562 (10.6) | | 1,617 (11.0) | | 1,640 (11.2) | 6,244 (10.6) |
|  | Mountain | 1,146 (7.8) | 1,142 (7.8) | | 1,033 (7.0) | | 1,036 (7.1) | 4,357 (7.4) |
|  | Pacific | 2,176 (14.8) | 1,699 (11.6) | | 1,647 (11.2) | | 1,293 (8.8) | 6,815 (11.6) |
| **Metropolitan/urban/rural ^a^, N (%)** | | | | | |  | |  |
|  | Metropolitan | 12,896 (87.9) | 12,732 (86.7) | | 12,552 (85.5) | | 12,054 (82.1) | 50,234 (85.5) |
|  | Urban and Rural | 1,783 (12.1) | 1,949 (13.3) | | 2,127 (14.5) | | 2,626 (17.9) | 8,485 (14.5) |

Note:

^a^ Metropolitan: population more than 250,000; Urban and rural: population less than 250,000.

Figure S1. Sample Selection

Figure S2. Sensitivity analysis. Comparison of results when weekly SSB price promotion for each household is defined as experiencing a weekly price magnitude X ≥5% vs. ≥2%, ≥10%, ≥15% ^a, b^

Association between household annual price promotion frequency and per capita purchase


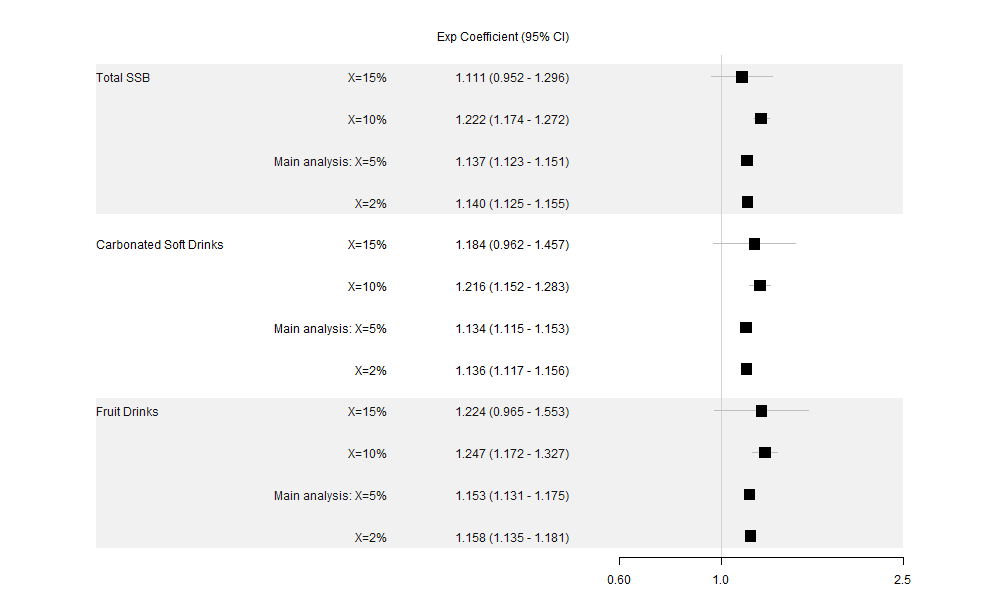


Notes:

^a^ Ordinary least squares linear regression was used. The outcome, annual per capita purchase, was log transformed. The models adjusted for household size, household income per capita, male head age, female head age, male head education, female head education, presence of children, race, male head occupation, female head occupation, region, and urban/rural setting.

^b^ Annual promotion frequency was calculated as: $\frac{Weeks experiencing \geq5\% magnitude of price promotion for the household}{Number of weeks in 2016}$.

Figure S3. Sensitivity analysis. Comparison of results when sample inclusion criteria is defined as purchased ≥80% of SSBs from stores in retailer database vs. purchased Y≥60%, ≥70%, or ≥90% of SSBs from stores in the retailer database ^a, b, c^

A. Association between household annual price promotion frequency and per capita purchase


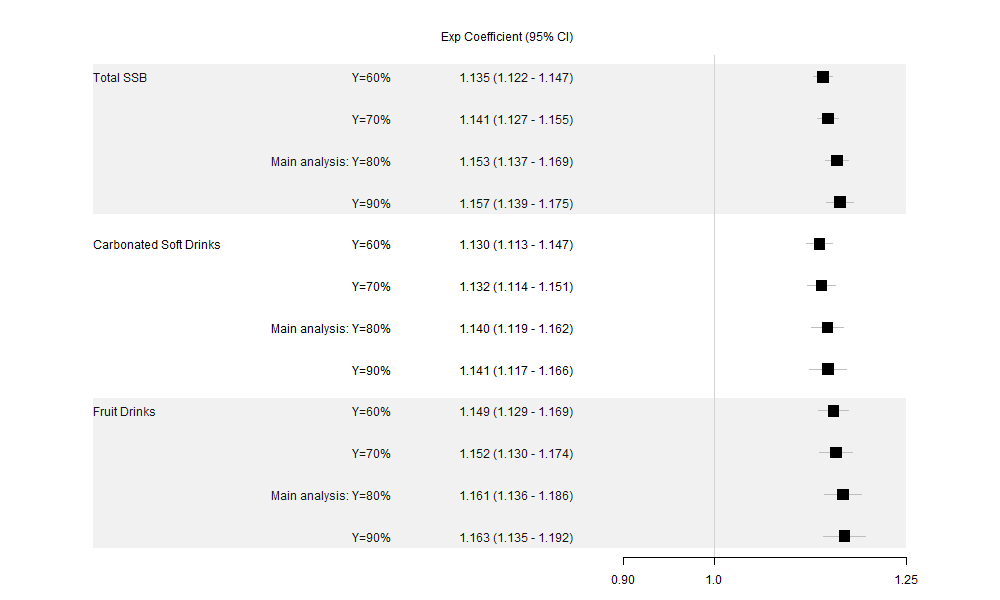


B. Association between household annual price magnitude and per capita purchase


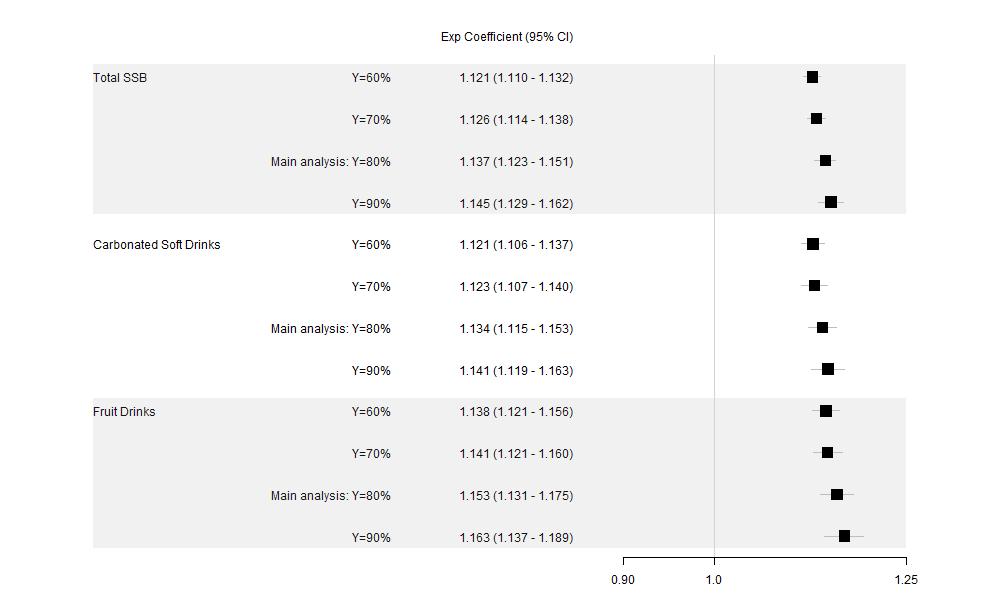


Notes:

^a^ Ordinary least squares linear regression was used. The outcome, annual per capita purchase, was log transformed. The models adjusted for household size, household income per capita, male head age, female head age, male head education, female head education, presence of children, race, male head occupation, female head occupation, region, and urban/rural setting.

^b^ Annual promotion frequency was calculated as: $\frac{Weeks experiencing \geq5\% magnitude of price promotion for the household}{Number of weeks in 2016}$

^c^ Annual promotion magnitude was calculated as: $\frac{\sum_{i=1}^{number of weeks in 2016} Promotion magnitude for the household in week i}{Number of weeks in 2016}$.

**Supplement References**

1. Brimblecombe J, Ferguson M, Chatfield MD, et al. Effect of a price discount and consumer education strategy on food and beverage purchases in remote Indigenous Australia: a stepped-wedge randomised controlled trial. *Lancet Public Health.* 2017;2(2):e82-e95.

2. Phipps EJ, Kumanyika SK, Stites SD, Singletary SB, Cooblall C, DiSantis KI. Buying food on sale: a mixed methods study with shoppers at an urban supermarket, Philadelphia, Pennsylvania, 2010-2012. *Prev Chronic Dis.* 2014;11:E151.

3. The Kilts Center. Nielsen Retail Scanner Dataset Manual. 2016.

4. Hendel I, Nevo A. Sales and consumer inventory. *The RAND Journal of Economics.* 2006;37(3):543-561.
